# Clinically-observed FOXA1 mutations upregulate *SEMA3C* through transcriptional derepression in prostate cancer

**DOI:** 10.1038/s41598-024-57854-w

**Published:** 2024-03-25

**Authors:** Kevin J. Tam, Liangliang Liu, Michael Hsing, Kush Dalal, Daksh Thaper, Brian McConeghy, Parvin Yenki, Satyam Bhasin, James W. Peacock, Yuzhuo Wang, Artem Cherkasov, Paul S. Rennie, Martin E. Gleave, Christopher J. Ong

**Affiliations:** 1 Vancouver Prostate Centre, Vancouver General Hospital, Vancouver, BC Canada; 2 Department of Urologic Sciences, University of British Columbia, Vancouver, BC Canada; 3 Department of Experimental Therapeutics, BC Cancer Agency, Vancouver, BC Canada

**Keywords:** Prostate cancer, Cancer genomics, Transcriptional regulatory elements

## Abstract

FOXA1 is a pioneer transcription factor that is frequently mutated in prostate, breast, bladder, and salivary gland malignancies. Indeed, metastatic castration-resistant prostate cancer (mCRPC) commonly harbour FOXA1 mutations with a prevalence of 35%. However, despite the frequent recurrence of FOXA1 mutations in prostate cancer, the mechanisms by which FOXA1 variants drive its oncogenic effects are still unclear. Semaphorin 3C (SEMA3C) is a secreted autocrine growth factor that drives growth and treatment resistance of prostate and other cancers and is known to be regulated by both AR and FOXA1. In the present study, we characterize FOXA1 alterations with respect to its regulation of SEMA3C. Our findings reveal that FOXA1 alterations lead to elevated levels of SEMA3C both in prostate cancer specimens and in vitro. We further show that FOXA1 negatively regulates SEMA3C via intronic cis elements, and that mutations in FOXA1 forkhead domain attenuate its inhibitory function in reporter assays, presumably by disrupting DNA binding of FOXA1. Our findings underscore the key role of FOXA1 in prostate cancer progression and treatment resistance by regulating SEMA3C expression and suggest that SEMA3C may be a driver of growth and tumor vulnerability of mCRPC harboring FOXA1 alterations.

## Introduction

FOXA1, a member of the forkhead box family of transcription factors, acts as a pioneer factor that binds to and de-compacts condensed chromatin to facilitate access of other transcription factors and transcriptional machinery to initiate the transcriptional cascade^1–6^. FOXA1 is well-documented to influence AR activity and can dramatically affect its cistrome^7–10^ leading to the up- or down-regulation of a large number of genes. However, the relationship between the AR and FOXA1 is complex. In prostate cancer, FOXA1 is known to have AR-dependent and AR-independent activities^10,11^; moreover, in those that are AR-dependent, FOXA1 can exert both positive and negative effects on expression of AR-regulated genes^11–13^. The nuances of FOXA1-AR interplay are likely dependent on biological context as well as proximity and arrangement of genomic FOXA1 motifs in relation to AR motifs.

Landmark genomic studies have revealed that FOXA1 is among the most frequently-mutated genes in prostate cancer patients^9,14–17^. These mutations have been documented in multiple independent clinical cohorts and associated with poor prognosis suggesting that *FOXA1* lesions may be a biomarker with potential diagnostic or prognostic significance. Recent coinciding reports have begun to unravel the clinical importance of FOXA1 mutations in PCa^18,19^. Adams et al. identified two mutation hotspots in *FOXA1* and demonstrated that FOXA1 variants trigger phenotypic changes by promoting growth and inducing epithelial-to-mesenchymal transition (EMT). Parolia et al. define three unique classes of FOXA1 variants that behave differently with respect to its effects on FOXA1 transcriptional activity and cell growth. However, molecular mechanisms by which FOXA1 variants mediate these tumor-promoting activities remain poorly understood.

To address this gap, we identified semaphorin 3C (SEMA3C) as a candidate FOXA1 target gene that might mediate the pro-oncogenic effects such as increased proliferation and EMT linked with FOXA1 variants in prostate cancer. SEMA3C is a secreted member of the semaphorin family of extracellular signaling proteins that has been implicated in various cancers including prostate, breast, gastric, lung, and pancreatic cancer, as well as glioblastoma^20–27^. We have previously shown that SEMA3C is regulated by both FOXA1 and AR in prostate cancer^28^. Furthermore, SEMA3C and its receptor, Plexin B1, drive cancer growth by transactivating multiple receptor tyrosine kinases including EGFR, HER2 and MET^20,29–31^. Additionally, SEMA3C has been linked to the induction of EMT and stemness of prostate epithelial cells as well as increased prostate cancer invasion and migration^21,29,32^. Importantly, since FOXA1-altered prostate cancer cell lines [AR-dependent LAPC4 (class-2 frameshift) and AR-independent DU145 (FOXA1-low)] display a dependence on SEMA3C for proliferation, this suggests that cancer cells bearing FOXA1 alterations may exhibit a tumor vulnerability and growth pathway addiction to SEMA3C. Collectively, these findings suggest that SEMA3C may be a putative driver of the cancer-promoting activities associated with FOXA1 variants, implying that the FOXA1 alterations commonly observed in clinical prostate cancer samples might mediate their oncogenic effects via SEMA3C.

To extend these findings we sought to better understand FOXA1’s control over *SEMA3C* expression. Given repeated observations of FOXA1 mutations in prostate cancer, we were also motivated to investigate the implications of clinically-observed mutations in FOXA1 on *SEMA3C* expression. Functional validation of genomic alterations to FOXA1 may be of clinical significance. Our results indicate that alterations to FOXA1 lead to increased *SEMA3C* expression levels in prostate cancer specimens and also in vitro. Additionally, we show that FOXA1 is negative regulator of *SEMA3C* expression through intronic elements, and that missense mutations in FOXA1 alter its transcriptional activities in reporter assays. We posit that these effects may be operationally due to modified protein-DNA interactions in FOXA1 mutants.

## Results

### FOXA1 alterations correlate with elevated *SEMA3C* expression in cBioPortal datasets

**Figure 1:**
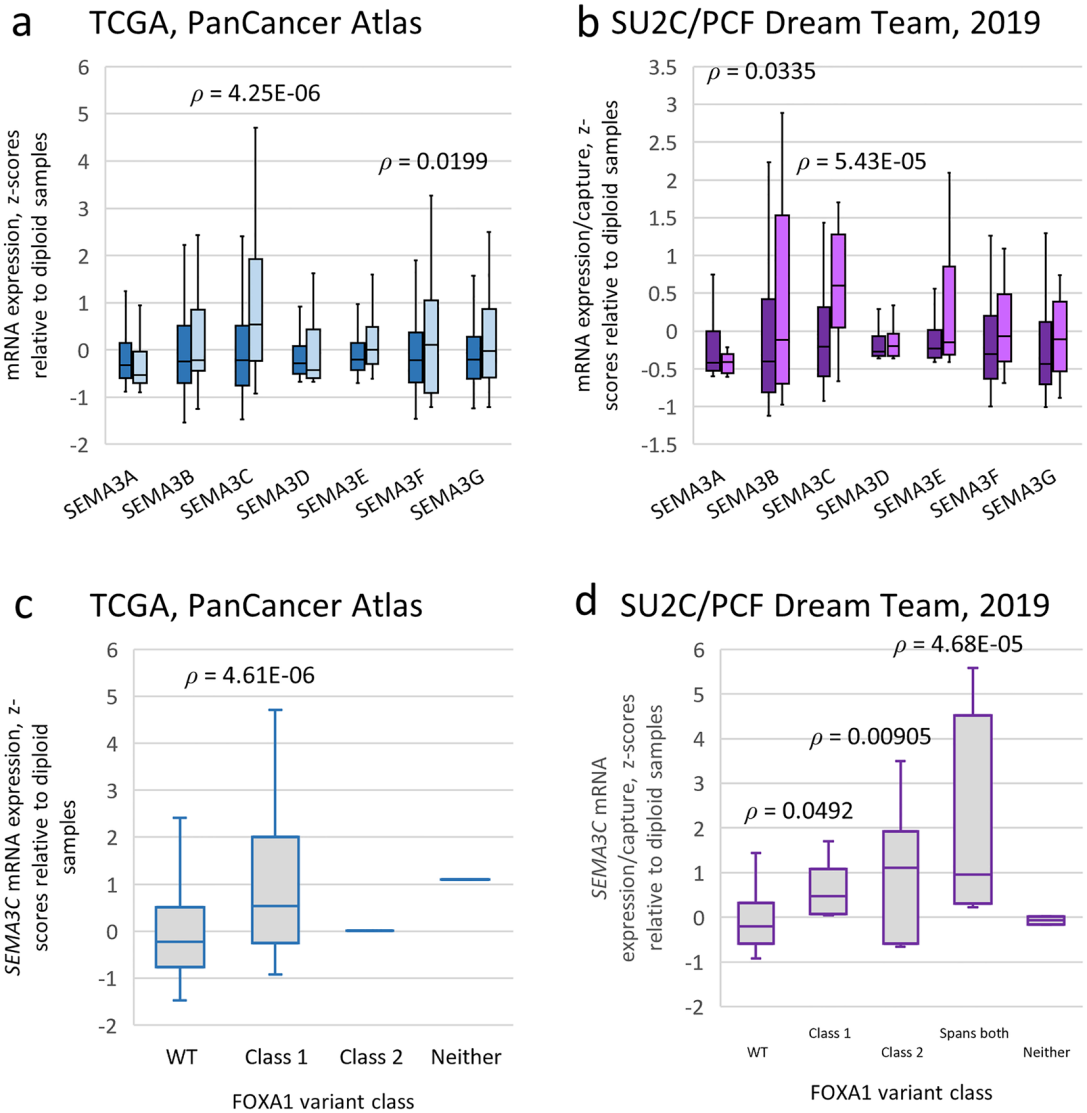
Class 3 semaphorin expression levels in wild-type versus altered FOXA1 prostate cancer specimens using cBioPortal. Boxplots of class 3 semaphorin mRNA levels in wild-type (shaded dark) versus altered FOXA1 specimens (shaded light) from the Prostate Adenocarcinoma cohort (TCGA, PanCancer Atlas) (**a**) and the Metastatic Prostate Adenocarcinoma cohort (SU2C/PCF Dream Team, PNAS 2019) (**b**) obtained from cBioPortal. Boxplots of class 3 semaphorin mRNA levels in the TCGA (**c**) and the SU2C/PCF (**d**) cohorts as a function of FOXA1 class variants. Class-1 variants included those in the FKHD (residues 168 to 269); class-2 variants included all variants c-terminal to residue 269; ‘Spans both’ in the SU2C/PCF cohort includes one or more variants that span residues used to define both class-1 and class-2 variants; ‘Neither’ indicates a variant in *FOXA1* that was outside of the definition of both class-1 and class-2 variants. The number of samples are as follows: for TCGA, WT n = 461, Class 1 n = 25, Class 2 n = 1, Neither n = 1; for SU2C/PCF, WT n = 187, Class 1 n = 9, Class 2 n = 6, Spans both n = 4, Neither n = 2. Boxes span the interquartile range. Horizontal line within the box represents the median SEMA3C mRNA levels. Bars represent the 95th percentile range of readings. Statistical analysis was performed using the unpaired two-tailed Student’s t-test relative to WT samples. TCGA, mRNA expression z-scores relative to diploid samples (RNA Seq V2 RSEM). SU2C/PCF 2019, mRNA expression z-scores relative to diploid samples (FPKM capture).

Considering FOXA1’s known control over *SEMA3C* expression combined with *FOXA1*’s predisposition to genomic alterations, we set out to determine if these two phenomena displayed any correlation. Using publicly-available TCGA and SU2C/PCF expression datasets, class 3 semaphorin levels in *FOXA1*-wild-type and *FOXA1*-altered (missense, insertions, deletions) specimens were compared (Fig. 1). This investigation revealed that, in aggregate, patients with *FOXA1* alterations have elevated levels of SEMA3C compared to their non-altered counterparts. Of note, among all class 3 semaphorins, differential expression between wild-type and altered *FOXA1* was most pronounced in *SEMA3C*. Parolia et al. classified class-1 FOXA1 mutations as those confined to the FKHD and class-2 as those c-terminal to the FKHD. When stratified in this way, *SEMA3C* expression was found to be elevated in both class-1 and class-2 variants compared to WT (Fig. 1c,d). The expression of other androgen receptor targets in wild-type and altered *FOXA1* samples within the TCGA and SU2C/PCF cohorts were also examined (Supplementary Figure S1).

### FOXA1 mutants alter the chromatin at the *SEMA3C* locus

**Figure 2:**
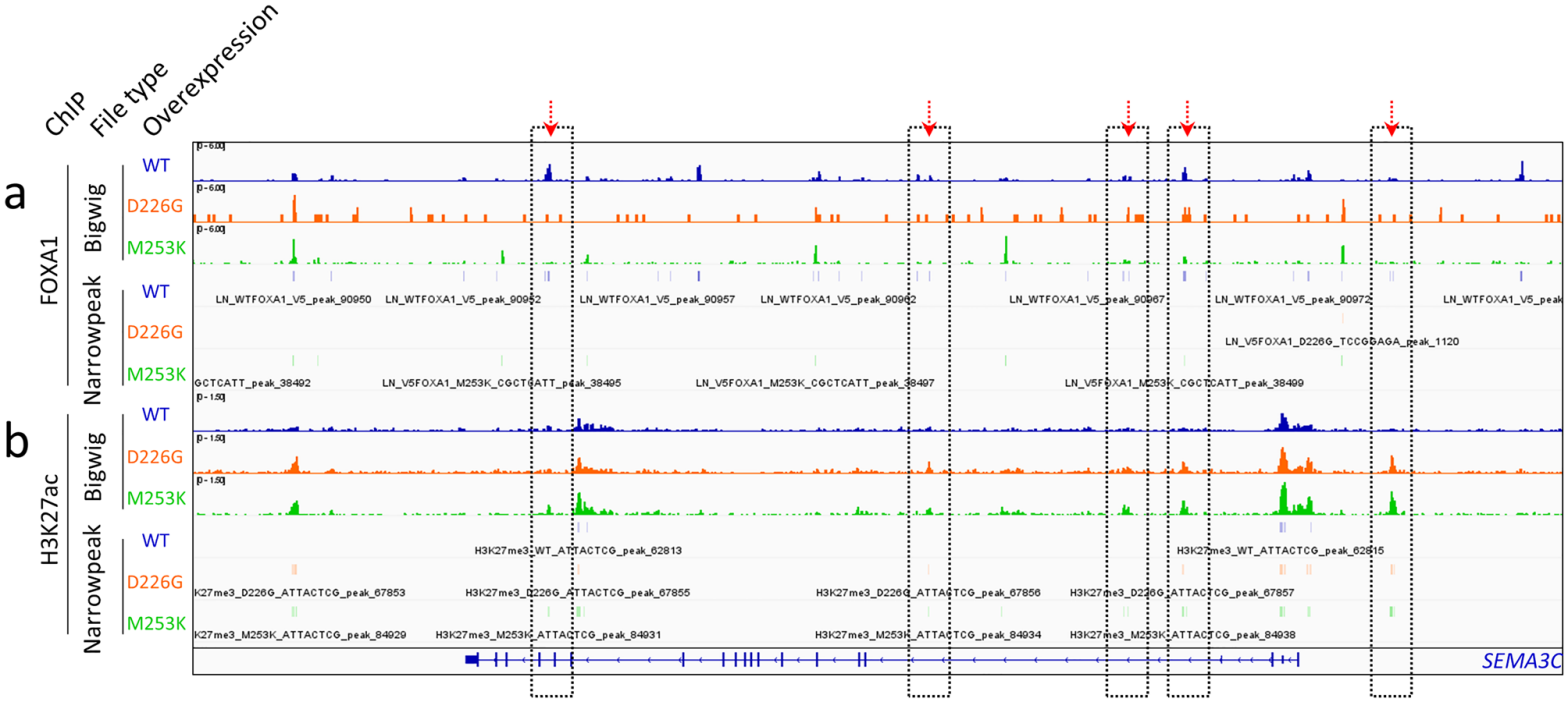
FOXA1 and H3K27ac ChIP-Seq in FOXA1 wild-type versus altered cells at the *SEMA3C* locus. To identify differential recruitment of FOXA1 (**a**) and H3K27ac (**b**) to *SEMA3C* imposed by FOXA1 alterations, bigWig (upper) and narrowPeak (lower) ChIP-Seq files produced by Gao et al., were examined in Integrative Genomics Viewer. FOXA1 and H3K27ac ChIP-Seqs in wildtype (blue), FOXA1 p.D226G (orange), and FOXA1 p.M253K (green) are displayed as indicated. The SEMA3C gene is shown below. Y-axes are fixed at 6.0 for the FOXA1 bigWig files and 1.5 for the H3K27ac bigWig files. Regions with a particularly dramatic shift in chromatin constituents between WT and variants are highlighted by red arrows and boxed.

To better understand if the tumor-level correlation between class-1 *FOXA1* alterations and *SEMA3C* expression is retained in in vitro systems, we first leveraged existing ChIP-Seq datasets from multiple recently-published reports. In a study by Gao et al., the authors overexpressed wild-type FOXA1 and the class-1 FOXA1 variants p.D226G and p.M253K in LNCaP and conducted FOXA1 ChIP-Seq (subseries GSE133386) or H3K27ac ChIP-Seq (subseries GSE133387). Examining this dataset using Integrative Genomics Viewer^33^ showed reduced recruitment of FOXA1 variants to the *SEMA3C* locus as compared to wild-type FOXA1 (Fig. 2a). This was concomitant with an increase in H3K27ac peaks in LNCaP overexpressing FOXA1 variants as compared to LNCaP overexpressing wild-type FOXA1 (Fig. 2b). Areas with pronounced redistribution of FOXA1 and H3K27ac at the SEMA3C locus between WT and variants are highlighted by red arrows and boxes. Taken together this would suggest that mutations to FOXA1 impair FOXA1’s interaction with the *SEMA3C* locus and increases acetylation marks on nearby histones. Adams et al. overexpressed wild-type Foxa1 and the class-1 Foxa1 variants p.F254_E255del and p.R219S in primary mouse organoids followed by FOXA1 or AR ChIP-Seq (subseries GSE128867). These too indicated differential recruitment of wild-type versus variants of FOXA1 to the *Sema3c* locus (Supplementary Figure S2). Parolia et al. overexpressed wild-type FOXA1 and the class-1 FOXA1 variants p.I176M and p.R261G in 22Rv1 cells followed by FOXA1 or AR ChIP-Seq (subseries GSE123618). These studies similarly showed reduced recruitment of FOXA1 to the *SEMA3C* locus by FOXA1 variants as compared to wild-type FOXA1 (Supplementary Figure S3). The impact that FOXA1 variants had on recruitment of AR to *SEMA3C* was inconsistent.

### FOXA1 mutants have altered gene expression

**Figure 3:**
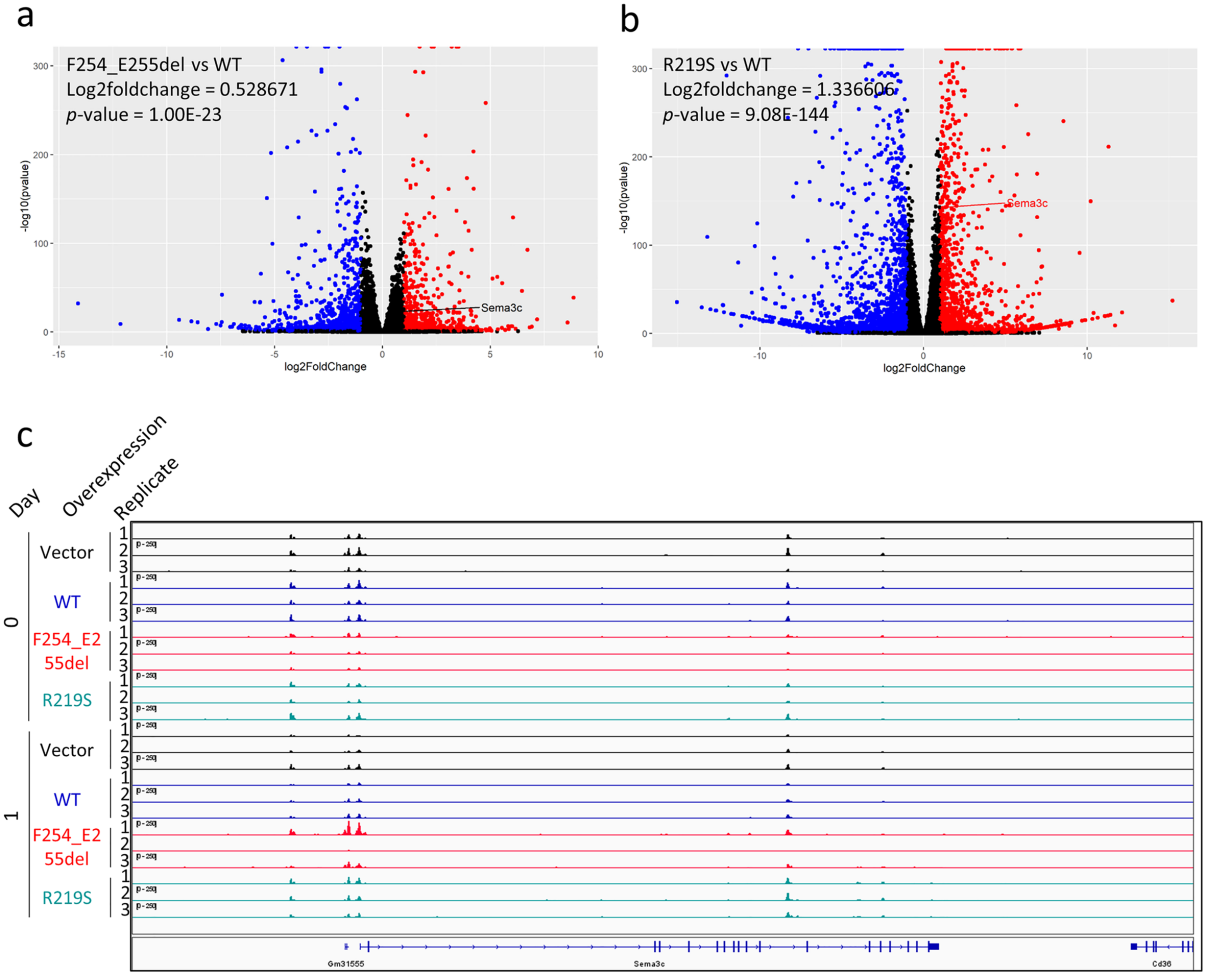
Global expression in murine organoids overexpressing Foxa1 constructs. Volcano plots comparing p.F254_E255del to WT (**a**) and p.R219S to WT (**b**) in the RNA-Seq data by Adams et al. (subseries GSE128666). X-axis is log2-fold change and y-axis is − log10 *p*-value. Day 1 triplicate data from Adams et al. was used. Expression is relative to wild-type Foxa1. Red is ≥ twofold upregulation and p < 0.05; blue is ≥ twofold downregulation and p < 0.05. The expression of Sema3c is indicated. Within the plot, Sema3c is highlighted. (**c**) ATAC-Seq bigWig files (subseries GSE128421) were visualized in Integrative Genomics Viewer. Day 0 and 1 in empty vector (black), wildtype (blue), Foxa1 p.F254_E255del (red), and Foxa1 p.R219S (cyan) are displayed. The Sema3c gene is shown below. Y-axes are fixed at 250.

Leveraging the RNA-Seq dataset by Adams et al. (subseries GSE128666), we were able to evaluate the effects of Foxa1 alterations on endogenous expression of *Sema3c*. In this study, Adams et al. stably transduced murine organoids with dox-inducible Foxa1 constructs or empty vector as control. We compared the *Sema3c* expression levels of the two Foxa1 variants (p.F254_E255del and p.R219S) to those of the wild-type line at the day 1 (of dox induction) time point. Overexpression of p.F254_E255del and p.R219S led to a 0.53 and 1.34 log2-fold induction of *Sema3c*, respectively, compared to wild-type Foxa1 (Fig. 3a,b). The associated *p*-values for p.F254_E255del and p.R219S were 1 × 10^−23^ and 9 × 10^−144^, respectively. These variants were also associated with states of more accessible Sema3c chromatin in ATAC-Seq analyses (subseries GSE128421; Fig. 3c). Examining the RNA-Seq data by Gao et al. (subseries GSE133384) revealed that the RPKM for *SEMA3C* changed only modestly in the p.D226G and p.M253T variants compared to wild-type (data not shown). Collectively, this work supports the notion that in in vitro systems, alterations to FOXA1 impart functional changes with regard to its regulation of *SEMA3C*.

### FOXA1 and AR DNA motifs overlap at the intron 2 of human *SEMA3C*

**Figure 4:**
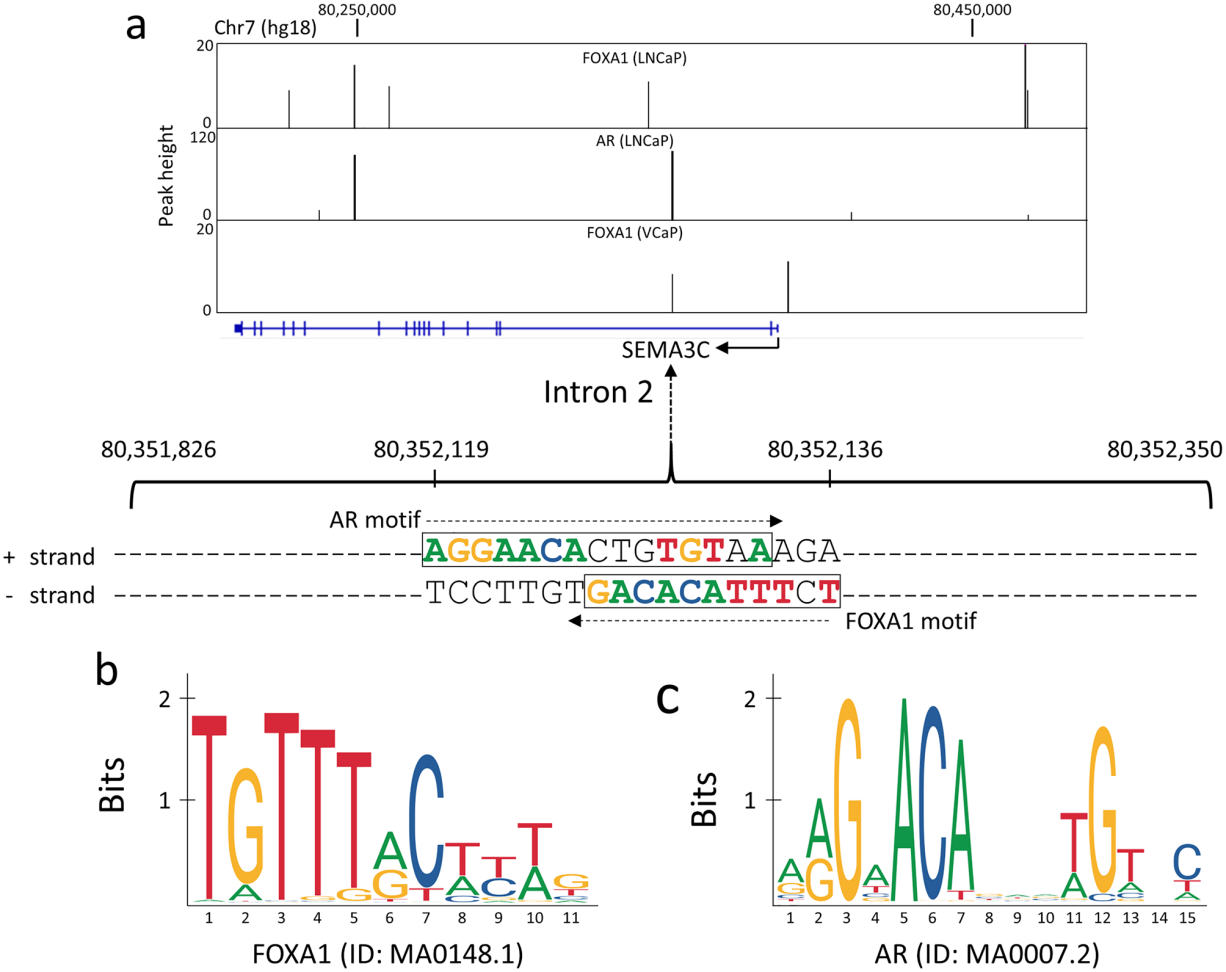
FOXA1 and AR DNA motifs at the human SEMA3C locus. (**a**) The ChIP-Seq peak data derived from the three experiments on FOXA1 in LNCaP, AR in LNCaP and FOXA1 in VCaP cells^34^ are shown as vertical black bars overlaid on the SEMA3C locus (blue horizontal line: exons are shown as vertical blue bars) using the UCSC Genome Browser (hg18, chromosome 7). The DNA sequences containing the FOXA1 and AR motifs, as predicted by the RSAT and JASPAR programs, are shown within the peak that spans from genomic positions 80,351,826 to 80,352,350 within intron 2 of SEMA3C. DNA sequences that match the consensus motifs are colored accordingly. The AR motif is located on the positive ( +) strand, and the FOXA1 motif is located on the minus (−) strand, both shown in a rectangular box respectively. (**b**) A consensus FOXA1 motif as reported in the JASPAR database is shown by a sequence logo. (**c**) A consensus AR motif as reported in the JASPAR database is shown by a sequence logo.

We previously demonstrated that the AR regulates *SEMA3C* expression through an androgen response element (ARE) located in intron 2 of *SEMA3C*^28^. The presence of this ARE was first identified in a ChIP-Seq study by Yu et al.^34^ that also reported a total of 35,107 and 11,774 genomic regions (or peaks) bound by the FOXA1 protein in LNCaP cells and VCaP cells, respectively. As illustrated in Fig. 4a, two such ChIP-Seq peaks are located at the intron 2 of *SEMA3C* on chromosome 7. The DNA sequences within these ChIP-Seq peaks were searched for the presence of FOXA1 (Fig. 4b) and AR DNA motifs (Fig. 4c), using two different motif scanning programs, RSAT^35^ and JASPAR^36^. A FOXA1 DNA motif was found within the ChIP-Seq peak at intron 2 (*p* = 6.4e−04), and an AR DNA motif was found within the same peak (*p* = 2.5e−05) through RSAT. Both the FOXA1 and AR motifs were also identified by the JASPAR program as the highest scoring motifs in that ChIP-Seq DNA region with relative scores of 0.88 and 0.94, respectively. The AR motif on the positive strand and the FOXA1 motif on the minus strand overlap on the same DNA region that spans from 80,352,119 and 80,352,136 (Fig. 4). We postulated that these DNA motifs were in some way responsible for regulation of *SEMA3C* expression in specimens containing FOXA1 variants.

### FOXA1 acts through a motif in the second intron of *SEMA3C*

**Figure 5:**
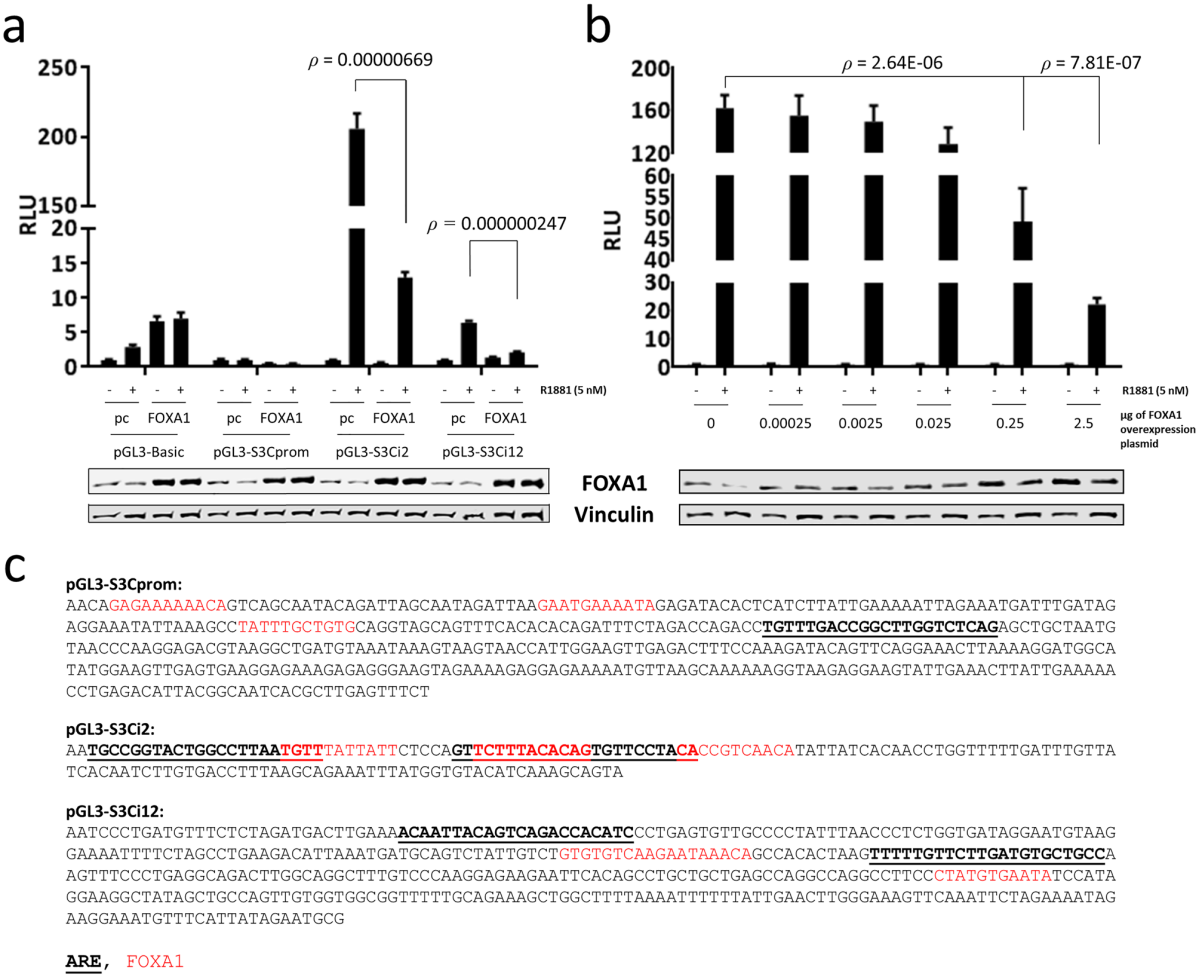
Cis elements in the FOXA1-binding regions of the *SEMA3C* locus. FOXA1 motif-containing regions of the *SEMA3C* promoter, second intron, and twelfth intron were cloned into the reporter vector, pGL3-Basic. LNCaP were co-transfected with reporter plasmids and either empty overexpression vector (‘pc’) or a FOXA1 overexpression plasmid (‘FOXA1’), stimulated with R1881 (5 nM), and then harvested to determine luciferase activity (**a**). Overexpression of FOXA1 was verified by Western blot. pGL3-S3Ci2 reporter construct was titrated against increasing amounts of FOXA1 overexpression plasmid (**b**). Western blot was used to monitor FOXA1 levels. Cloned regions are displayed and FOXA1 motifs (red) and AR elements (bold, underlined) are indicated (**c**). Minus strands are shown.

To ascertain which cis element(s) within the *SEMA3C* locus might be regulated by FOXA1, FOXA1-binding regions in the promoter, intron 2, and intron 12 were cloned into luciferase reporter vectors to generate pGL3-S3Cprom, pGL3-S3Ci2, and pGL3-S3Ci12, respectively. Cloned sequences are displayed along with top-ranked FOXA1 motifs and AREs as determined by RSAT and JASPAR (Fig. 5c). LNCaP were co-transfected with the reporter vector and FOXA1 overexpression plasmids and then treated with R1881. Overexpression of FOXA1 profoundly suppressed R1881-induced induction of pGL3-S3Ci2 and, to a lesser extent, pGL3-S3Ci12 (Fig. 5a). Neither R1881 nor FOXA1 overexpression affected transactivation of pGL3-S3Cprom or empty vector (pGL3-Basic) to the same degree as pGL3-S3Ci2. Overexpression of FOXA1 was verified by Western blot (Fig. 5a, lower). LNCaP were co-transfected with pGL3-S3Ci2 and increasing amounts of the FOXA1 overexpression plasmid and then treated with R1881. These experiments showed that FOXA1-mediated attenuation of R1881-induced transcription is dose-dependent (Fig. 5b). Sequence analysis of the second intron of murine *Sema3c* showed a stretch with high similarity to the region in the second intron of human *SEMA3C* containing the FOXA1 and AR motifs (Supplementary Figure S4). Collectively, these findings align with our previous observations indicating that FOXA1 is a potent negative regulator of *SEMA3C* expression^28^ and suggest that FOXA1 operates through cis elements located in the second intron of *SEMA3C*.

### FOXA1 variants have reduced transcriptional repressive activity in reporter gene assays

**Figure 6:**
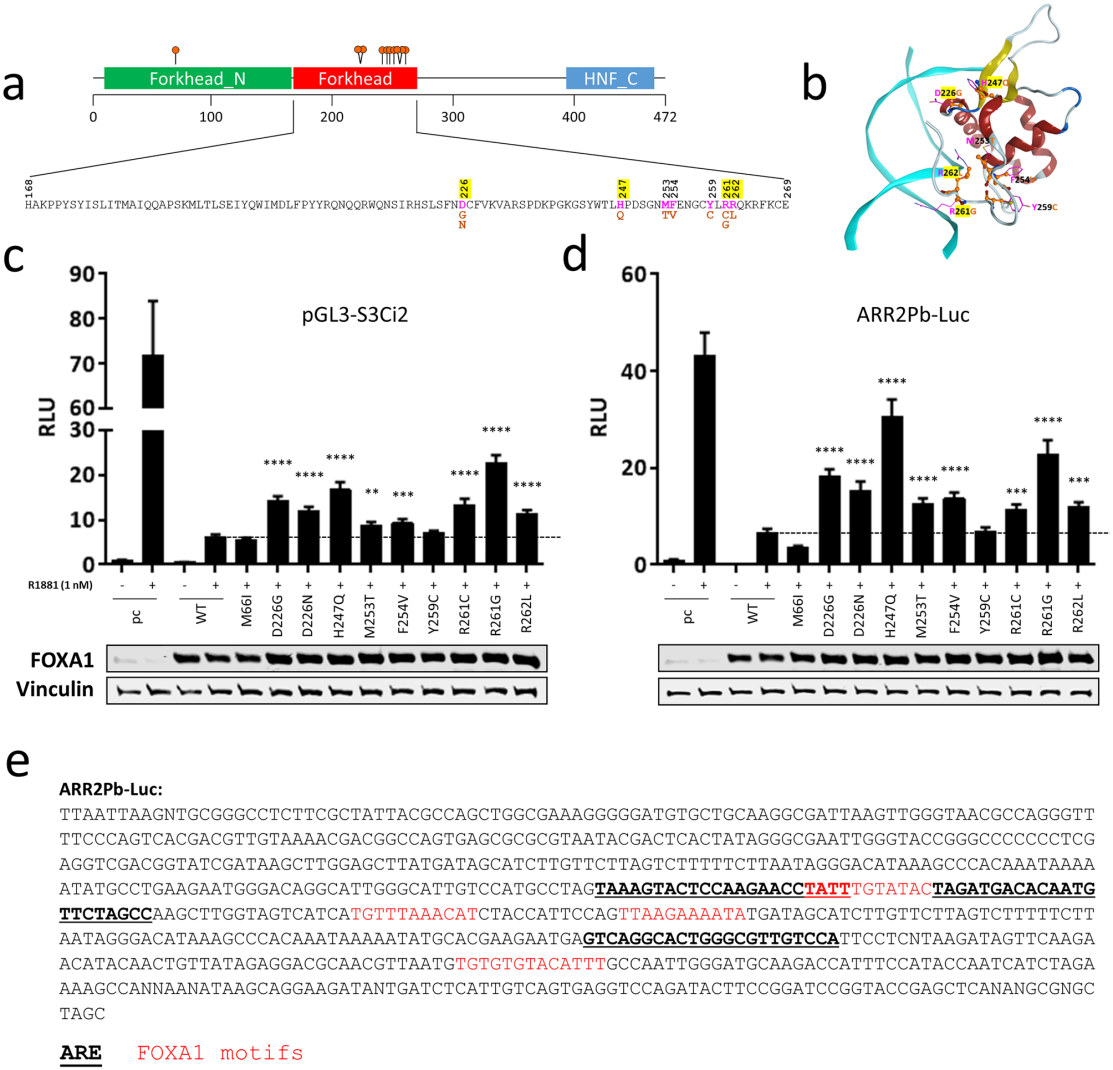
The effect of FOXA1 variants on androgen-responsive reporter constructs. (**a**) Schematic illustration of the FOXA1 gene with amino acid substitutions chosen for this study shown below. Nine of the constructs generated had a mutation at the c-terminus of the FKHD; one construct, M66I, contained a mutation n-terminal to the FKHD DNA-binding domain. Charged residues are highlighted in yellow. (**b**) Numerous mutations selected for our studies are located at the FOXA1-DNA interface. Amino acid changes, as a result of DNA mutations investigated in this study, are shown on the forkhead domain of the FOXA1 protein (alpha-helix in red and beta-strand in yellow ribbons). DNA is shown in cyan ribbons. Wild-type amino acids are shown in pink, with substitutions shown in orange. To determine the impact of missense mutations to FOXA1 in reporter gene assays, wild-type (WT) and FOXA1 mutants were co-transfected into cells along with AR-responsive reporter vectors for 2 days and then stimulated with R1881 in 0.2% CSS Opti-MEM for 24 h (1 nM) for an additional 24 h. Reporter constructs used included pGL3-S3Ci2 which harbours the intronic *SEMA3C* androgen response element (**c**) and ARR2Pb-Luc (**d**). Empty vector (pcDNA3.1 or ‘pc’) was transfected as a negative control. Relative Luminescence Units (RLU, y-axis) was scaled to sample pc without R1881, which was set to 1. Luminescence was read on a TECAN Infinite M200. Data represent mean, ± SD; **p < 0.01, ***p < 0.001, ****p < 0.0001. Significance is compared to sample: WT + R1881 (horizontal bar) by a Student’s t-test. Overexpression was confirmed by Western blot analysis; vinculin served as a loading control. (**e**) The cloned sequence within ARR2Pb-Luc is shown with AREs and FOXA1 motifs indicated in bold/underline and red, respectively. The sequence within pGL3-S3Ci2 was shown previously.

We next set out to understand the functional impacts of FOXA1 mutations in our reporter assays. While many *FOXA1* mutations have been annotated to date, nine class-1 variants and one non-class-1 variant were selected for this study as illustrated schematically in Fig. 6a. Notably, numerous FOXA1 mutants investigated in this study contain amino acid substitutions located at the interface between the FOXA1 forkhead domain and DNA (Fig. 6b). These substitutions are predicted to weaken the protein-DNA interactions as residues such as Arg261 and Arg262, which are known to interact with the nucleotides and DNA backbone^37^, have been changed to non-interacting Gly261 and Leu262, respectively. To functionally characterize the FOXA1 mutants, we employed reporter gene assays using the pGL3-S3Ci2 construct due to its robust response in Fig. 5a. When FOXA1 constructs were co-transfected with pGL3-S3Ci2, wild-type FOXA1 suppressed R1881-induced luciferase activity whereas the missense mutations: D226G, D226N, H247Q, R261C, R261G, and R262L, showed impaired suppression compared to wild-type FOXA1 (Fig. 6c). This was also the case when another androgen-responsive construct, ARR2Pb-Luc, was used. Indeed, wild-type FOXA1 suppressed R1881 induction of luciferase activity but mutants D226G, D226N, H247Q, M253T, F254V, R261C, R261G, and R262L showed decreased suppression compared to wild-type FOXA1. M66I and Y259C showed enhanced and no altered suppression, respectively (Fig. 6d). Cloned sequence in ARR2Pb-Luc is displayed along with top-ranked FOXA1 motifs and AREs (Fig. 6e). Through modelling, we note that those variants whose mutated residues face the DNA have the most profound loss of activity (Fig. 6b). In particular, mutations from charged residues to hydrophobic ones were associated with the biggest effects. Furthermore, mutations at charged residues that reside in close proximity to the DNA impacted FOXA1’s transcriptional activity the greatest, possibly by hindering DNA binding. We acknowledge the fact that reporter assays present a limited view of the endogenous events that take place during gene expression and that events like chromatin re-modelling and pioneering events, which also influence gene expression, are not properly captured in reporter assays. Nonetheless, our data demonstrate that alterations to FOXA1 have functional consequences and may be responsible for altered *SEMA3C* expression in prostate cancer specimens.

## Discussion

Interrogation of clinical datasets revealed that patients with FOXA1 alterations have higher *SEMA3C* expression levels. Similar findings were discovered using in vitro ChIP-Seq, RNA-Seq, and ATAC-Seq datasets. Our reporter assays indicate that FOXA1 negatively regulates *SEMA3C* by way of cis elements in the second intron of *SEMA3C*. Furthermore, the variants D226G, D226N, H247Q, R261C, R261G, and R262L seemingly diminished FOXA1’s repressive effects leading to increased luciferase activity in constructs bearing FOXA1 motifs. FOXA1’s inhibitory effects on AR in reporter assays may relate to DNA occupancy by FOXA1 given the fact that ChIP-Seq analyses showed reduced binding of FOXA1 mutants to the *SEMA3C* locus. Moreover, point mutations mapping to the DNA-binding residues of FOXA1 forkhead domain strongly affected its repressive activity.

Our work adds to the body of knowledge amassing around FOXA1 in prostate cancer. We demonstrate that at least some of the clinically-observed FOXA1 alterations have functional consequences and, aided by computer modelling, infer that the structural basis for functional changes are related to altered protein-DNA interaction. These findings add support to the existing belief that FOXA1 mutations have tangible and measurable repercussions. Undoubtedly, biological context will influence the manifestation of each variant. Whether these mutations drive disease progression and the clinical interpretations of FOXA1 variants remains to be fully understood.

Adams et al. and Parolia et al. illuminate numerous clinical and biological ramifications of mutations to FOXA1 in PCa. In agreement with our findings, Adams et al. show that mutations to the residues D226, H247, and R261 are among the most impactful to FOXA1 in reporter assays compared to wild-type FOXA1. Our work is further substantiated by Parolia et al. who show that the R261G variant has more potent AR-driven transcriptional activity than wild-type FOXA1. Interestingly, Adams et al. discovered the enrichment of a non-canonical FOXA1 binding motif, resembling the GTAAA(C/T) motif but with a substitution of (G/A) for (C/T) at position 6, among FOXA1 p.R219S ChIP-Seq and ATAC-Seq peaks. The FOXA1 motif in intron 2 of *SEMA3C* conforms to the non-canonical motif opening up the possibility that variants of FOXA1 p.R219 play a unique role in *SEMA3C* regulation.

The data presented here builds on existing work and shows that elevated SEMA3C levels in FOXA1-alterated prostate cancer patients may be linked to intronic FOXA1 elements in *SEMA3C*. We note that the FOXA1 motif in the second intron of *SEMA3C* overlaps with the ARE (Fig. 4) which we previously determined to confer positive AR regulation of *SEMA3C*. It is therefore possible that FOXA1 normally co-occupies this ARE or displaces AR from this region with the net effect of dampened *SEMA3C* expression. If *FOXA1* incurs a mutation, then FOXA1’s repression of AR-mediated *SEMA3C* expression may be lifted giving rise to elevated *SEMA3C* expression and acquisition of cell motility, invasiveness, and mesenchymal or stem-like phenotypes. Further analyses are warranted to prove that the proposed interaction between FOXA1 and AR at the second intron of *SEMA3C* is actually occurring and whether or not this particular type of interplay between FOXA1 and AR occurs elsewhere in the genome. It should also be noted that while we have drawn a link between FOXA1, AR, and SEMA3C in prostate cancer, a direct causal relationship cannot be concluded without further studies.

Work presented here outlines one avenue of *SEMA3C* regulation and is intended to inspire future work which will be needed to fully understand the mechanisms governing elevated SEMA3C levels in prostate cancer patients containing *FOXA1* alterations. Our reporter assays are a measure of transcription initiation, but numerous other mechanisms likely underpin heightened *SEMA3C* expression which may or may not include cis-elements examined in our investigations. As an example, a notable FOXA1 ChIP-Seq peak upstream of *SEMA3C* should also be investigated for its roles in regulating *SEMA3C* expression. In addition, rescue experiments should be conducted to determine if a wild-type FOXA1 cistrome at *SEMA3C* can be restored at peaks lost due to *FOXA1* mutations. Also, despite its effects on *SEMA3C* chromatin, overexpression of *FOXA1* variants in LNCaP did not alter *SEMA3C* expression based on findings by Gao et al. and also in our own hands (data not shown). This indicates that the functional impacts of mutations to *FOXA1* in luciferase assays alone, do not account for changes in *SEMA3C* expression or that endogenous expression of wild-type FOXA1 may mask changes induced by ectopic expression of *FOXA1* variants. Additional studies will be needed to fully dissect the details and extent of FOXA1 regulation of *SEMA3C*. In particular, interrupting endogenous FOXA1 through genome editing, rather than through exogenous FOXA1, would be highly informative. Yet other future studies to carry out include examining other variants of the same residue (eg H247Q vs. H247R). In addition, numerous other missense mutations have been annotated in FOXA1 and should also be characterized. This work sets the precedent for the regulation of semaphorins by FOXA1 and the potential effects of FOXA1 mutations in this context. As FOXA1 is seen to be mutated in other forms of cancer, such as breast cancer, mutational analysis of FOXA1 should be examined in this area as well.

The multifaceted role of FOXA1 in regulating gene expression highlights the distinction between oncogenic driver mutations and tumor suppressor loss-of-function mutations. While FOXA1 is traditionally recognized as a pioneer factor that enhances gene transcription, our findings show it can also act as a repressor for certain growth-promoting genes. In particular, FOXA1 seems to restrain the expression of the SEMA3C growth factor by binding to its intronic cis elements. Intriguingly, class-1 variants of FOXA1 appear to dampen this repressive action. This dynamic may help reconcile seemingly divergent roles of FOXA1 as both an oncogene and a tumor suppressor. Our data, which highlights FOXA1's inhibitory influence on the SEMA3C growth pathway and the gain-of-function activities of FOXA1 mutations that diminish its repressive activities on SEMA3C, offers a framework that bridges these two contrasting views.

## Materials and methods

### Bioinformatics and ChIP-Seq data analysis

Previous ChIP-Seq data from Yu et al.^34^ was extracted from the NCBI Gene Expression Omnibus (GEO; RRID:SCR_005012) database^38^. In particular, the file (‘GSM353633_20A9RAAXX_C425.allregions.txt.gz’), which contained 35,107 DNA regions (i.e. peaks) bound by the FOXA1 protein in LNCaP cells (GEO accession: GSM353633), was parsed to a bedGraph format and visualized in the UCSC Genome Browser^39^ to identify FOXA1 binding sites nearby to the SEMA3C (RefSeq accession number NM_006379) locus on chromosome 7 of the human reference genome (hg18). Same analysis was done on the file (‘GSM353630_20A9RAAXX_C421.allregions.txt.gz’), which contained 11,774 DNA regions bound by the FOXA1 protein in VCaP cells (GEO accession: GSM353630), and the file (‘GSM353644_jy10s123.allregions.txt.gz’), which contained 44,536 DNA regions bound by the AR protein in LNCaP cells treated with R1881 (GEO accession: GSM353644). The actual DNA sequences that compose each binding peak region (~ 500 bps) at the SEMA3C locus were extracted and scanned for any presence of the FOXA1 (11 bps) and AR motif (15 bps), using DNA motif scanning programs, RSAT^35^ and JASPAR^36^. The DNA frequency matrices that define the FOXA1 motif (ID: MA0148.1) and AR motif (ID: MA0007.2) were obtained from the JASPAR database^36^. ChIP-Seq, RNA-Seq, and ATAC-Seq in FOXA1 wild-type versus variant-expressing cells was obtained at the GEO accession numbers: GSE133455, GSE128667, and GSE123625. bigWig and narrowPeak files were visualized in Integrative Genomics Viewer^33^ for ChIP-Seq and ATAC-Seq analyses. Differential gene expression analysis was carried out using DESeq2 (RRID:SCR_015687) using default parameters. Log2-fold expression is versus wild-type. Clustal Omega^40^ and ClustalW were used for sequence alignment.

### FOXA1 protein structure modeling

The forkhead domain of the human FOXA1 protein structure was modeled based on the same domain of human FOXA3 (PDB ID: 1VTN^37^) that shares 96% sequence identity across the 100 amino acids (residue #168 to #267). The four amino acid differences were modified to match the FOXA1 sequence (G188S, E209Q, A245T, S249D) using the MOE protein modeling tool^41^.

### DNA sequences

In reporter gene assays, genomic sequence encompassing highly-ranked FOXA1 motifs were cloned into the luciferase reporter backbone pGL3-Basic (Promega, E1751) to generate reporter constructs. Sequences are displayed in Fig. 5. Other AR-responsive reporter vectors were a kind gift from Dr. Paul S. Rennie (Department of Urologic Sciences, University of British Columbia). FOXA1 overexpression was achieved using a wild-type FOXA1 overexpression plasmid was obtained from Genscript (Clone ID: OHu23484, NM_004496.3). Mutant FOXA1 constructs were derived from this plasmid using QuikChange II Site-Directed Mutagenesis Kit (Stratagene, 200,523). pcDNA3.1 (RRID:Addgene_79663) served as an empty vector control. Plasmids were transfected using TransIT-2020 (Mirus, MIR 5404).

### Cell culture

LNCaP (ATCC, CRL-1740, RRID:CVCL_0395) were cultured in RPMI 1640 supplemented with 10% FBS. Cells were treated at the indicated concentrations of androgen or 0.05% ethanol as a vehicle control in 0.2% charcoal-stripped serum (CSS) in Opti-MEM (Gibco, 11,058–021).

### Luciferase assay

5 × 10^5^ LNCaP cells were transiently transfected in full serum-containing media in triplicate in 12-well format with 0.6 µg of reporter vector or reporter vector control and 0.6 µg of wild-type or mutant FOXA1 or empty overexpression plasmid control as well as 30 ng of renilla plasmid (phRL-SV40) kindly provided by the Mui lab (Immunity and Infection Research Centre, Vancouver Coastal Health Research Institute, Vancouver, British Columbia). Two days following transfection, unless otherwise stated cells were treated with EtOH or R1881 in 0.2% CSS Opti-MEM for 24 h at which time cell lysates were harvested for luciferase assay using the Dual-Luciferase Reporter Assay System (Promega, E1960) and read on a TECAN Infinite M200 PRO. ARR2Pb-Luc is an androgen-responsive construct containing the probasin promoter. In all luciferase assays, firefly luciferase luminescence was normalized to renilla luciferase luminescence.

### Western blot

Whole cell extracts were prepared in 50 mM Tris–HCl, 150 mM NaCl, 1% NP40, 10 mM NaF, 10% Glycerol, supplemented with protease inhibitor cocktail (Roche, 04,693,116,001) and quantitated using a BCA approach. 60 µg of protein was run on 10% acrylamide gels and transferred onto nitrocellulose membrane. Western blots were imaged on a LI-COR Odyssey system. Vinculin served as a loading control. Primary antibodies: FOXA1 (Santa Cruz Biotechnology, sc-6553, RRID:AB_2104865) and vinculin (Sigma-Aldrich, V4505, RRID:AB_477617). Secondary antibodies: anti-mouse alexa fluor 680 (Invitrogen, A21058, RRID:AB_2535724), and anti-goat alexa fluor 680 (Invitrogen, A21084, RRID:AB_2535741).

### Clinical datasets

The Prostate Adenocarcinoma cohort (TCGA, PanCancer Atlas) and Metastatic Prostate Adenocarcinoma cohort (SU2C/PCF Dream Team, PNAS 2019) were obtained from cBioPortal^42,43^. Gene expression in patients with mutated FOXA1 were compared to patients with unaltered FOXA1 within the same dataset.

### Statistical analyses

Statistical analysis was performed using the Student’s two-tailed t-test. Data are represented as mean ± SD unless otherwise stated. Data presented a representative of three biological replicates.

### Supplementary Information

Supplementary Figures.

## Data Availability

The data analyzed in this study were obtained from cBioPortal and Gene Expression Omnibus (RRID:SCR_005012) at GSM353633, GSM353630, GSM353644, GSE133455, GSE128667, and GSE123625.
