# Supplementary material for: Clinically-observed FOXA1 mutations upregulate SEMA3C through transcriptional derepression in prostate cancer
Source: Sci Rep. 2024 Mar 25;14:7082. doi: 10.1038/s41598-024-57854-w (PMC10963789; doi:10.1038/s41598-024-57854-w)
Supplement: Supplementary file 1 — Supplementary Figures. [file 41598_2024_57854_MOESM1_ESM.pdf]

Clinically-observed FOXA1 mutations upregulate *SEMA3C* through transcriptional derepression in prostate cancer

Kevin J. Tam[1], Liangliang Liu[1], Michael Hsing[1], Kush Dalal[1], Daksh Thaper[1,2], Brian McConeghy[1], Parvin Yenki[1,2], Satyam Bhasin[1,2], James W. Peacock[1,2], Yuzhuo Wang[1,2,3], Artem Cherkasov[1,2], Paul S. Rennie[1,2], Martin E. Gleave[1,2], and Christopher J. Ong[1,2*]

[1]Vancouver Prostate Centre, Vancouver General Hospital, Vancouver, BC, Canada

[2]Department of Urologic Sciences, University of British Columbia, Vancouver, BC, Canada

[3]Department of Experimental Therapeutics, BC Cancer Agency, Vancouver, BC, Canada

*Correspondence to: chris.ong@ubc.ca

Supplementary Figure S1.

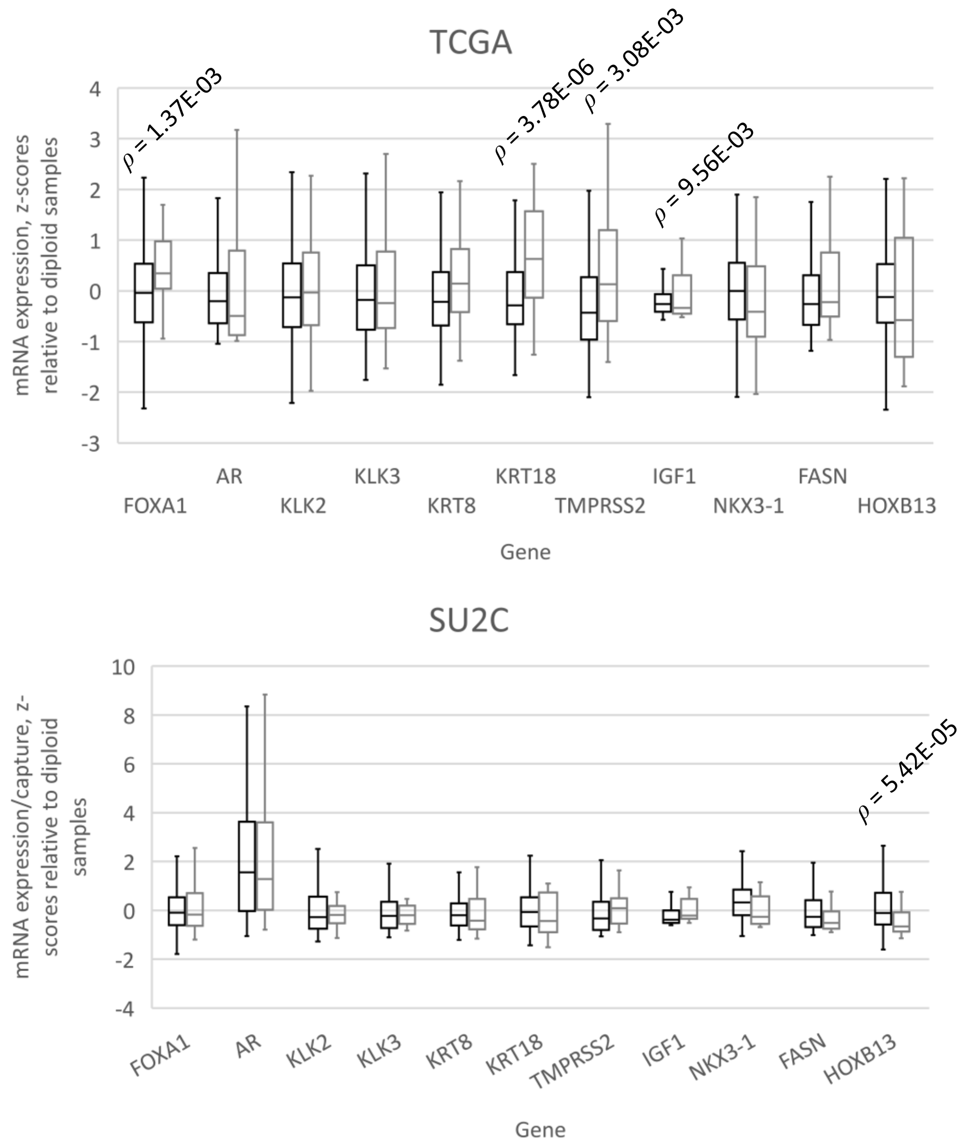

Supplementary Figure S1. Expression levels of androgen receptor targets in wild-type versus altered FOXA1 prostate cancer specimens. Boxplots of mRNA levels of the genes listed on the x-axis in wild-type (black) versus altered (grey) FOXA1 specimens from the Prostate Adenocarcinoma cohort (TCGA, PanCancer Atlas) and the Metastatic Prostate Adenocarcinoma cohort (SU2C/PCF Dream Team, PNAS 2019) obtained from cBioPortal. Boxes span the interquartile range. Horizontal line within the box represents the median mRNA levels. Bars represent the 95th percentile range of readings. Statistical analysis was performed using the unpaired two-tailed Student’s t-test relative to WT samples. TCGA, mRNA expression z-scores relative to diploid samples (RNA Seq V2 RSEM). SU2C/PCF 2019, mRNA expression z-scores relative to diploid samples (FPKM capture).

Supplementary Figure S2.

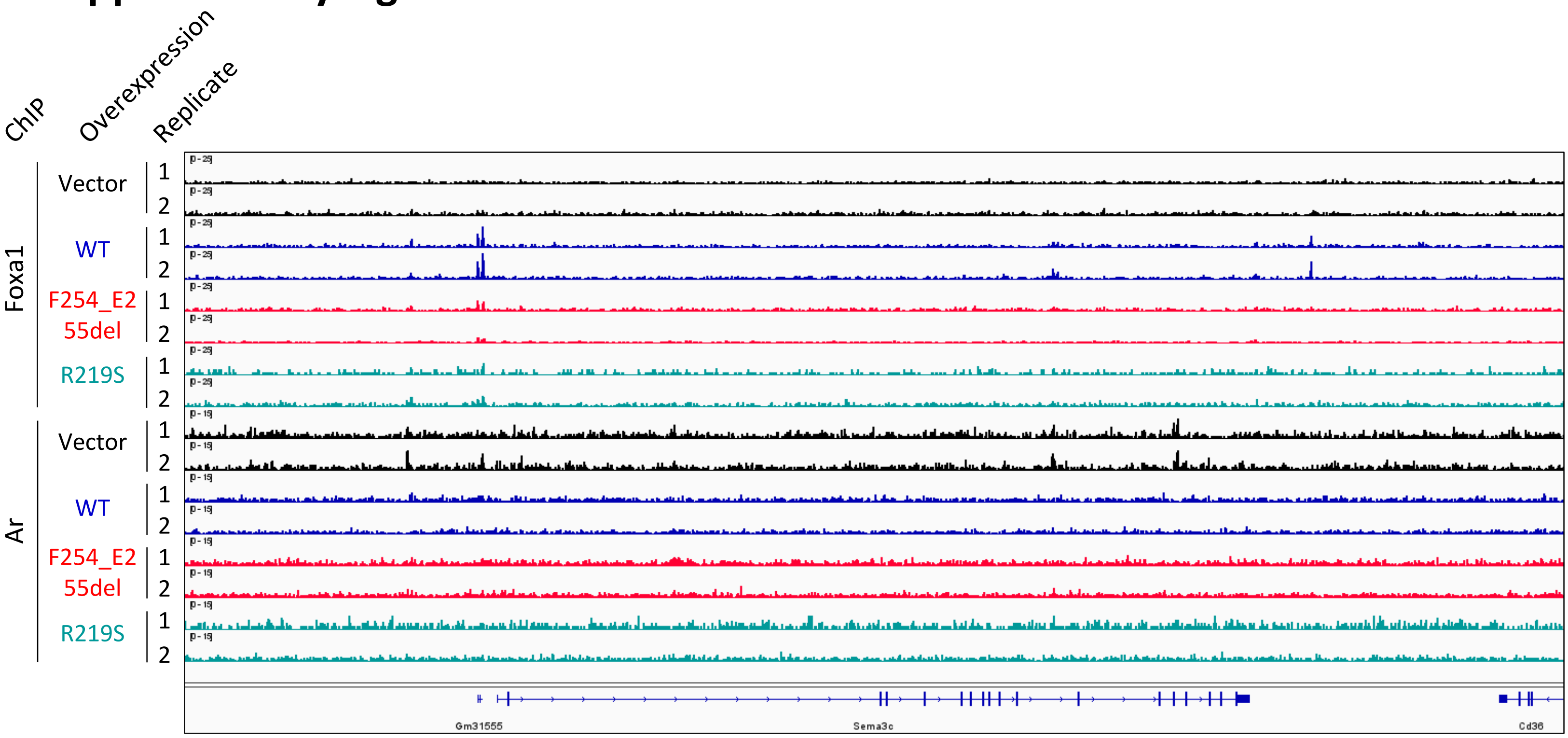

Supplementary Figure S2. FOXA1 and AR ChIP-Seq in FOXA1 wild-type versus altered cells at the *Sema3c* locus in primary mouse prostate organoids. Y-axis are fixed for the bigWig files.

Supplementary Figure S3.

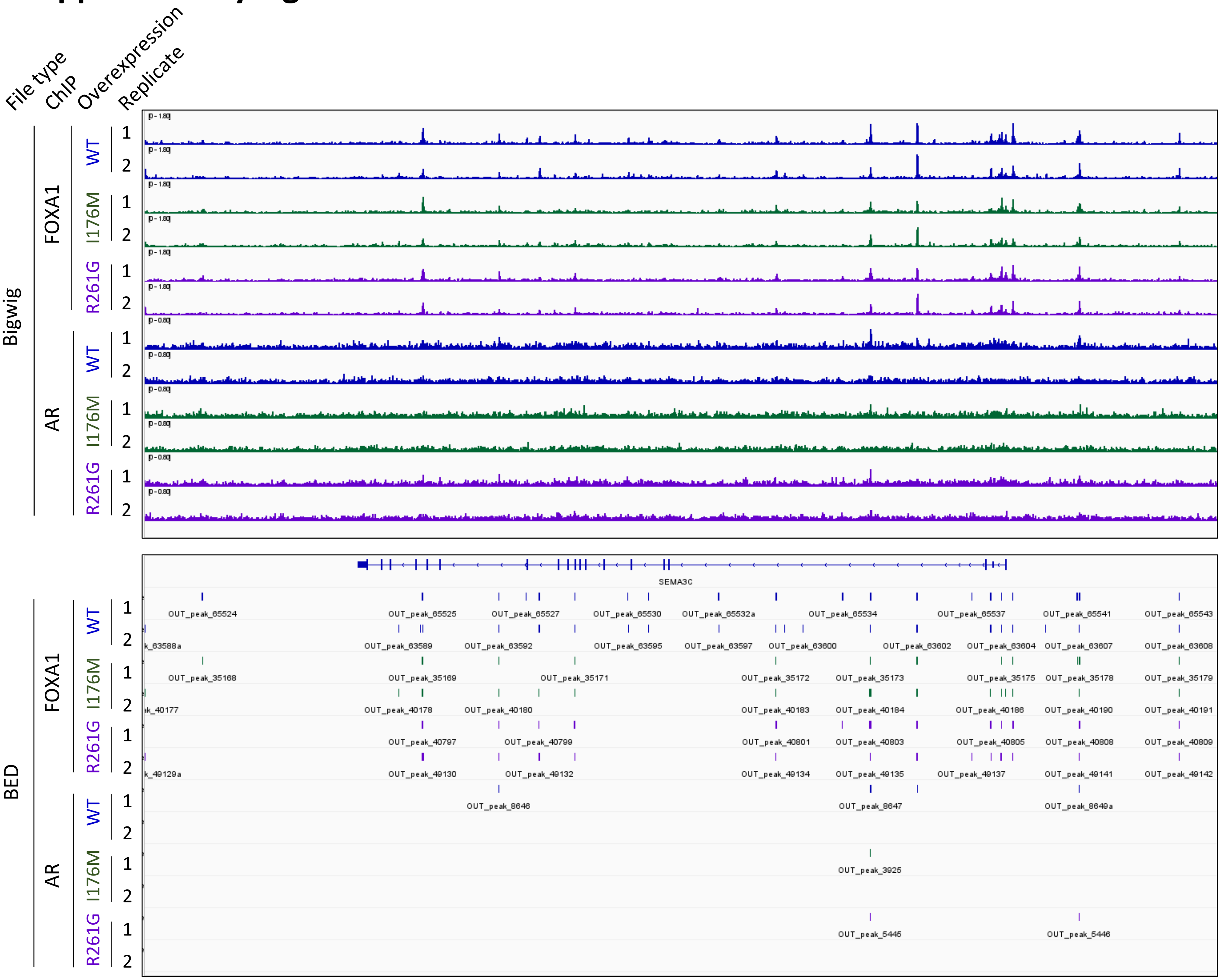

Supplementary Figure S3. FOXA1 and AR ChIP-Seq in FOXA1 wild-type versus altered cells at the *SEMA3C* locus in 22Rv1 cells. Y-axis are fixed for the bigWig files.

Supplementary Figure S4.

a

```
mm10_dna        AAGTACAGTTTATGGAAGCCTTGCATGGGAAGGATTTCATACAAAGGATAACTTCAGCAC
pGL3-S3Ci2      ------------------------------------------------------------

mm10_dna        AACATAAAACATTAGGTTGACACTTCCTGGGAACACAGGTTTTCTTAAAGTGTTTTCAAA
pGL3-S3Ci2      ------------------------------------------------------------

                                    chr5:17601762
                                             |
mm10_dna        GGTTTGCTTCTGTGTGTCGCTACATTGTTGCTACATATTCTGGTCTTAATGTTTATTATT
pGL3-S3Ci2      -----------------------------AATGCCGGTACTGGCCTTAATGTTTATTATT
                                               * *   * **** ****************

mm10_dna        CTCCAGTGCCTCAGACAGTTTTCCTGTATTATTAACATAT----ACAACCTGGGTTCTGA
pGL3-S3Ci2      CTCCAGTTCTTTACACAGTGTTCCTACACCGTCAACATATTATCACAACCTGGTTTTTGA
                ******* * * * ***** *****  *   * *******    ********* ** ***
                                                                           ┌chr5:17601908
mm10_dna        TTGTGTCATCGCAATCTGGTCACCTTTCAGCAGATATTTATGGTGGACATCAAAGCAGTC
pGL3-S3Ci2      TT-TGTTATCACAATCTTGTGACCTTTAAGCAGAAATTTATGGTGTACATCAAAGCAGTA
                ** *** *** ****** ** ****** ****** ********** *************

mm10_dna        ATAAACACATAAAGACATTCCTCTGCTCAGAAAAGCTCTCACTTTGCCGTTGTTATGGAA
pGL3-S3Ci2      ------------------------------------------------------------

mm10_dna        CATGTGGCCAGGGTTTACATCTGCAGTACCTCCCTCTAGTTTATTTGGAAAACAGTGCTG
pGL3-S3Ci2      ------------------------------------------------------------

mm10_dna        CCATTTGTAAGGGGTTCACTGCTTATATTCTCGCATCAACACTTTTCCCCCCAAGCAGTT
pGL3-S3Ci2      ------------------------------------------------------------
```

b

```
>mm10,chr5:17601762-17601908
GCTACATATTCTGGTCTTAATGTTTATTATTCTCCAGTGCCTCAGACAGTTTTCCTGTATTA
TTAACATATACAACCTGGGTTCTGATTGTGTCATCGCAATCTGGTCACCTTTCAGCAGATAT
TTATGGTGGACATCAAAGCAGTC
```

ARE, FOXA1

Supplementary Figure S4. Homology analysis of murine *Sema3c* locus. Using Clustal tools, the second intron of murine *Sema3c* (mm10, chr5:17,576,971-17,653,707) was aligned with the region in *SEMA3C* intron 2 containing FOXA1 and AR motifs, specifically, the sequence associated with pGL3-S3Ci2 shown in Figure 5C. (a) Sequence alignment showed strong similarity between the sequence in pGL3-S3Ci2 and chr5:17,601,762-17,601,908 of the second intron of *Sema3c*. (b) Motif analysis of this region showed putative FOXA1 and AR motifs.

Supplementary Information.

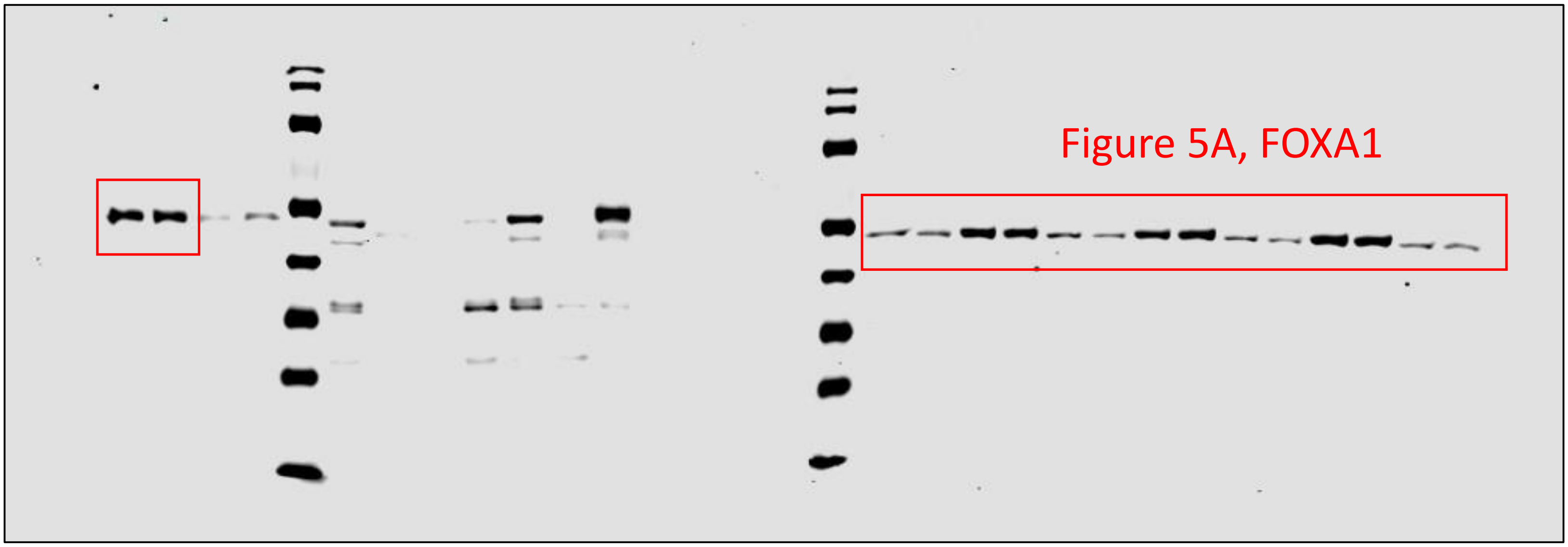

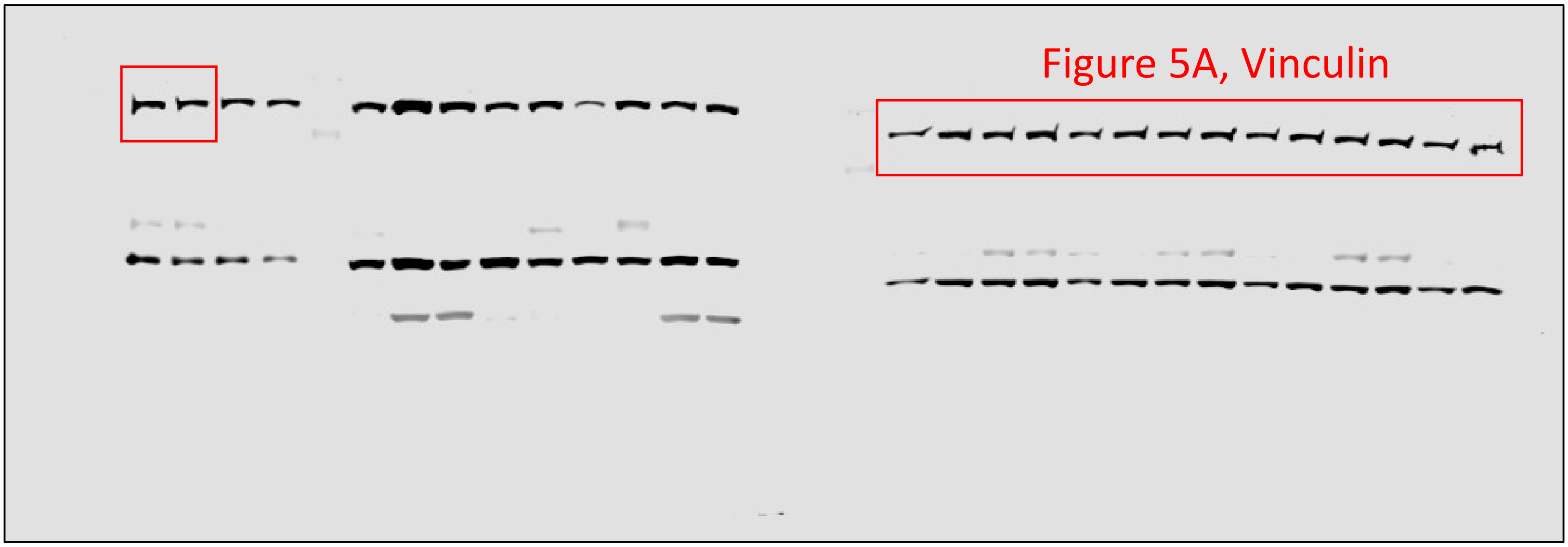

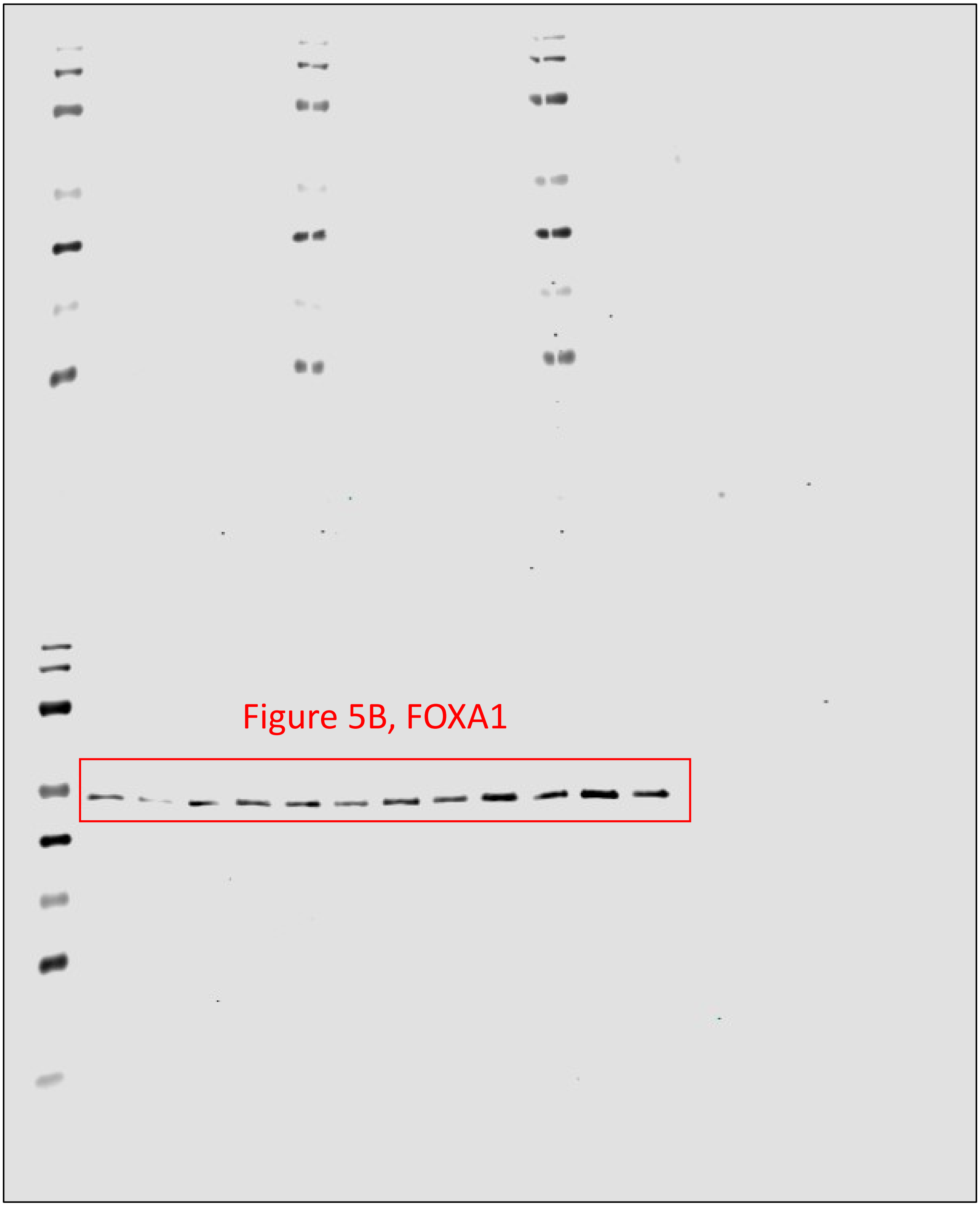
Figure 5B, FOXA1

Figure 5B, Vinculin

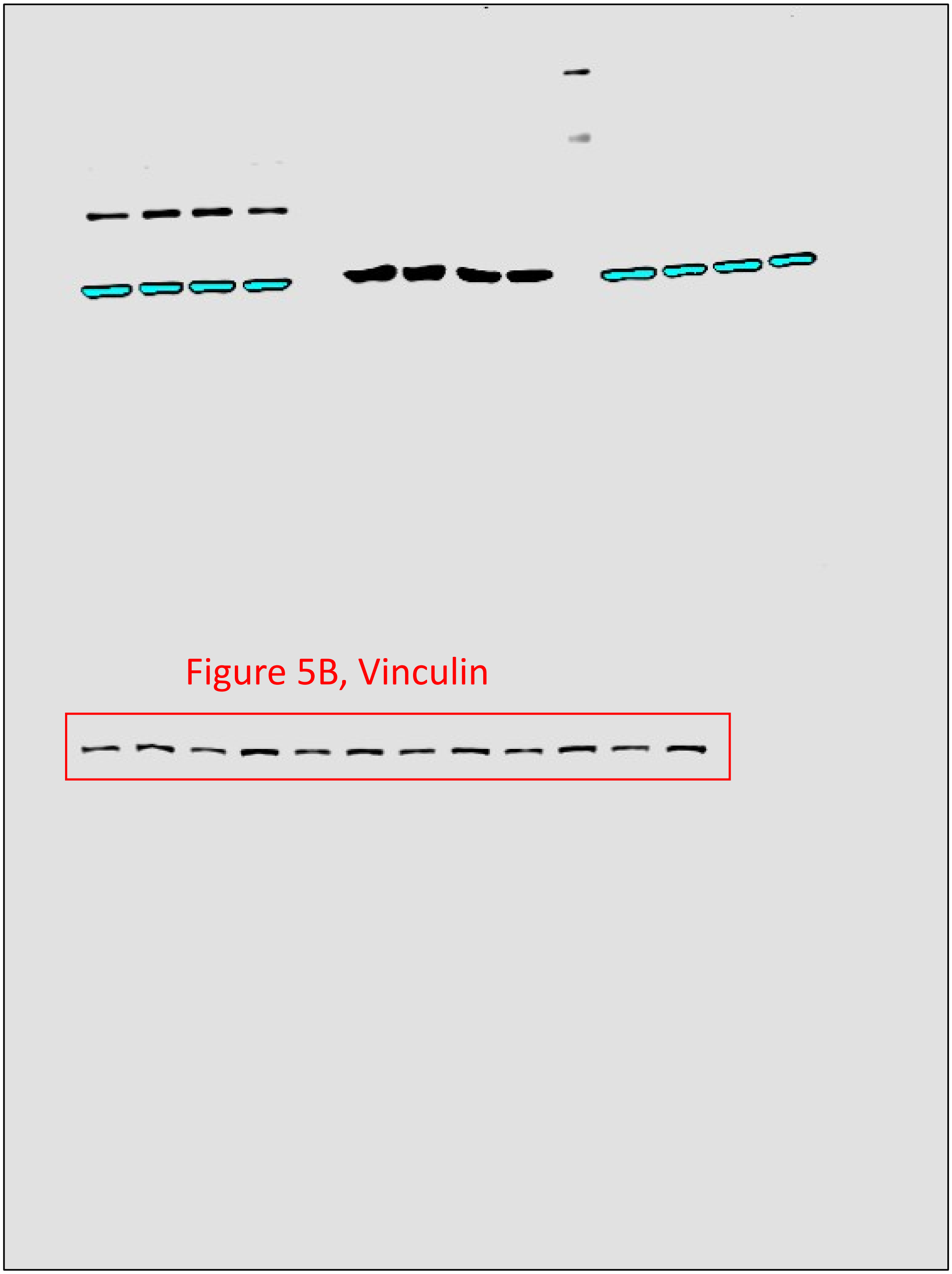

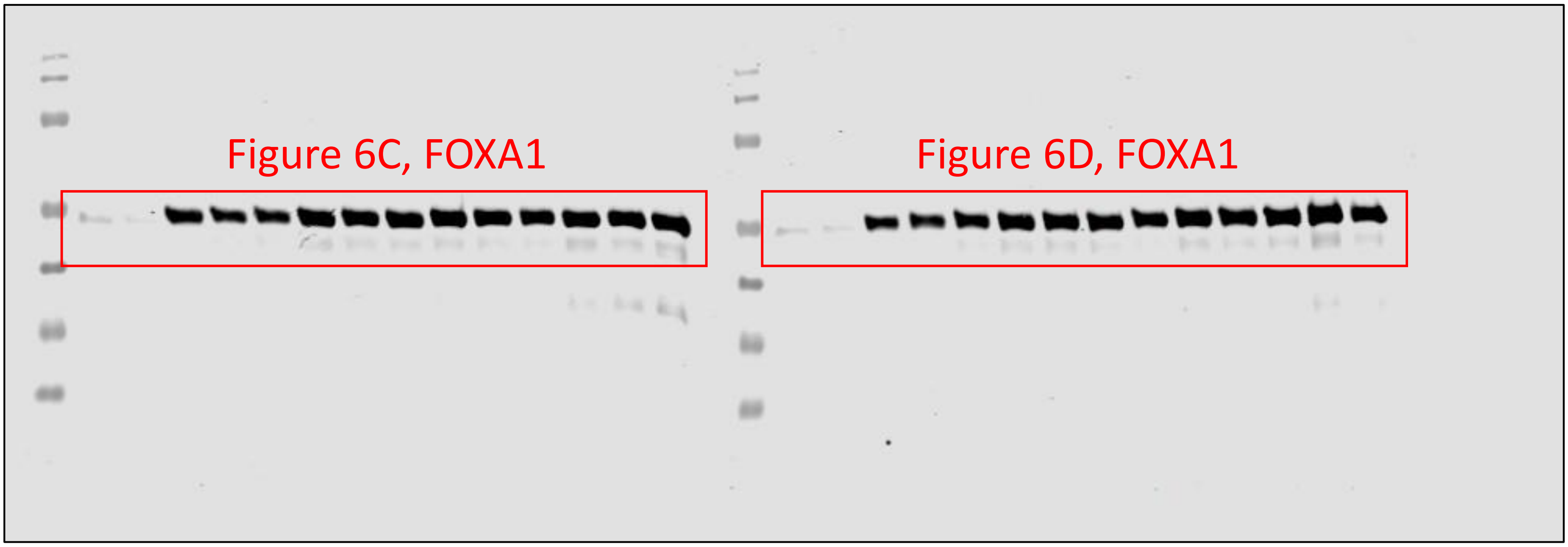

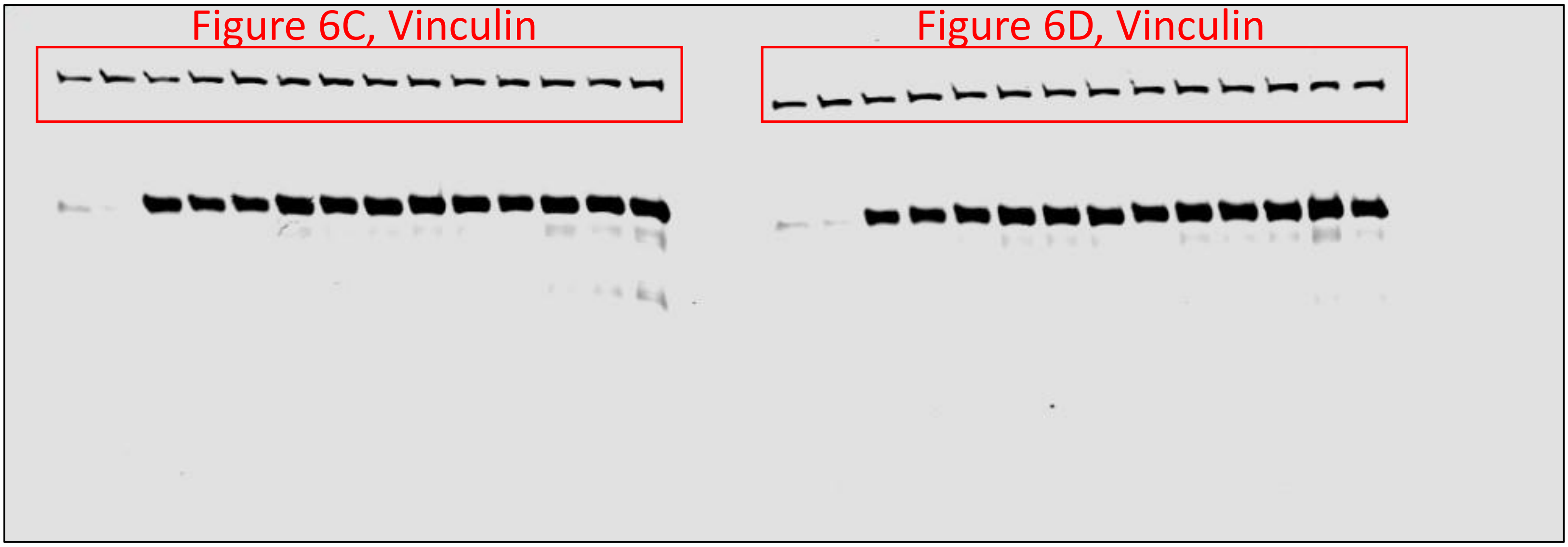

Supplementary Information. Uncropped Western blots. Uncropped Western blots from Figures 5 and 6 are provided. Each area boxed in black represents the area selected in the LI-COR Odyssey control software for a single scan, which contains multiple blots. Thermo Fisher Scientific PageRuler Prestained Protein Ladder 10 to 180 kDa (26616) was used in each case. The approximate area used for Figures 5 and 6 are indicated by red boxes. The specific panel in which the image was used is indicated in red text. For Figure 5A, two separate gels were necessary to accommodate the 16 samples due to limitations in the number of wells per acrylamide gel.
